# Effect of Different Luting Protocols on the Bond Strength of Fiber-Reinforced CAD/CAM Blocks

**DOI:** 10.3390/polym18020160

**Published:** 2026-01-07

**Authors:** Irem Buyukates, Sufyan Garoushi, Pekka K. Vallittu, Sadullah Uctasli, Lippo Lassila

**Affiliations:** 1 Department of Prosthetic Dentistry, Faculty of Dentistry, Baskent University, 06490 Ankara, Turkey; 2 Department of Biomaterials Science, Faculty of Dentistry, University of Turku, 20520 Turku, Finland; pekval@utu.fi (P.K.V.); liplas@utu.fi (L.L.); 3 Turku Clinical Biomaterial Center—TCBC, Institute of Dentistry, University of Turku, 20520 Turku, Finland; 4 Wellbeing Services County of Southwest Finland, 20521 Turku, Finland; 5 Department of Prosthetic Dentistry, Faculty of Dentistry, Ankara University, 06560 Ankara, Turkey; uctasli@dentistry.ankara.edu.tr

**Keywords:** CAD/CAM, fiber reinforced CAD composite, shear bond strength, luting resin

## Abstract

The aim was to evaluate the shear-bond strength (SBS) of experimental short fiber-reinforced CAD/CAM composites (SFRC-CAD) and commercial CAD/CAM composites (Cerasmart 270) to different luting resin composites before and after hydrothermal aging. Discs (2 mm) obtained from SFRC-CAD and Cerasmart 270 were air-particle abraded and treated with a primer (G-CEM One Enhancing Primer) with or without universal adhesive (G2 Bond). A fiber-reinforced flowable composite (everX Flow) and a self-adhesive resin cement (G-CEM One) were used as luting materials under direct or indirect curing conditions. Thirty-two experimental groups were determined based on restorative material, bonding protocol, luting resin, curing technique, and aging procedure (n = 8/group). SBS was measured after 24 h of water storage or following hydrothermal aging. Data were analyzed using nonparametric statistical tests (*p* < 0.05). No statistically significant differences in SBS were observed between everX Flow and G-CEM One regardless of the bond application (*p* > 0.05). SFRC-CAD bonded with everX Flow and universal adhesive demonstrated significantly higher SBS than the corresponding Cerasmart groups (*p* < 0.05), whereas no significant differences were observed between comparable groups when G-CEM One was used. Failure mode analysis showed predominantly adhesive and mixed failures, with no cohesive failures within SFRC-CAD. Overall, the everX Flow proved to be an effective luting material, indicating that this material may be suitable for luting CAD/CAM indirect restorations.

## 1. Introduction

Advances in digital dentistry have led to ceramic and ceramic-like CAD/CAM materials replacing metal-supported restorations due to their biocompatibility and superior mechanical and optical properties [1,2,3,4]. The biomimetic concept further supports using glass-matrix or hybrid ceramics for enamel replacement and composites, including short fiber-reinforced types, for dentin substitution [5,6,7]. Nevertheless, their inherent brittleness, relatively high cost, and technique-sensitive adhesive cementation procedures remain clinical challenges.

To address these challenges, resin composite CAD/CAM materials have been introduced as viable alternatives, combining ceramic-like mechanical behavior with dentin-like elasticity [8]. CAD/CAM resin composites exhibit improved properties; however, their ability to reinforce structurally compromised teeth remains limited [9].

To overcome the limitations of resin composite restorative materials in general and to reinforce structurally compromised teeth, fiber-reinforced composite resins have been introduced [10]. The incorporation of reinforcing fibers into polymer matrices has been shown to significantly enhance mechanical performance by enabling effective stress transfer, crack-bridging mechanisms, and improved resistance to deformation due to the high aspect ratio and load-bearing capacity of fibers. Unlike particulate fillers, fibers provide directional reinforcement while the surrounding polymer matrix ensures homogeneous distribution and protects the fibers from environmental degradation [11]. Initially incorporated into direct flowable and packable composites, the approach was adapted for CAD/CAM fabrication, resulting in short fiber-reinforced composite (SFRC-CAD) blocks for indirect restorations. The addition of randomly oriented glass fibers increases both flexibility and stiffness, resists crack initiation and propagation, and improves fracture toughness [12,13]. Recent studies have reported that experimental SFRC-CAD blocks demonstrated improved mechanical strength, resulting in increased load-bearing capacity and favorable surface characteristics [12,14,15].

As with all indirect restorations, their long-term success depends not only on the restorative material itself but also on the performance of the luting agent. Resin-based cements are preferred due to their low solubility, minor water uptake, and durable adhesion to both tooth tissues and restorative substrates when used in conjunction with contemporary adhesive systems [3,16,17,18]. Self-adhesive resin cements further simplify the procedure by eliminating separate etching or priming steps, although their adhesion can be enhanced by combining them with primers or adhesive resins when required [19,20]. The success of the adhesive cementation procedure depends not only on the adhesive system and resin-based luting material used, but also on surface treatments and conditioning [21,22,23].

Polymerization efficiency is another decisive factor in the success of adhesive cementation. Light transmission through CAD/CAM materials is limited due to optical attenuation, which reduces cement polymerization [24,25,26]. Significant light attenuation can reduce the degree of polymerization, limiting the hardness, wear resistance, and biocompatibility of resin cements [27,28]. While light-cured cements generally achieve higher degree of monomer conversion than chemically cured ones, the extent of curing in clinical conditions depends heavily on the optical properties of the restorative material [29]. Although dual-cure resin cements incorporate a chemical curing component, light activation remains a critical contributor to achieving optimal polymerization and mechanical properties, particularly beneath indirect restorations.

Despite the increasing use of SFRC-CAD blocks in laboratory studies, evidence remains limited on how curing mode, cement type and adhesive strategy affect bonding performance compared with hybrid ceramics. This study provides a systematic evaluation of the bonding behavior of an experimental short fiber-reinforced CAD/CAM composite compared with a commercial CAD/CAM material, with particular emphasis on the influence of luting material selection, curing strategy, and accelerated hydrothermal aging. In addition, the use of a fiber-reinforced flowable composite as a luting material is explored as a potential alternative to conventional resin cements. Therefore, this study investigated the effects of two light-curing techniques (direct light exposure and light exposure through the restoration), two types of luting cements (fiber-reinforced composite and self-adhesive resin), and two CAD/CAM restorative materials (hybrid ceramic and SFRC-CAD) on shear bond strength. The null hypothesis was that these factors would not significantly affect bond strength.

## 2. Materials and Methods

Two CAD/CAM restorative materials—a short fiber-reinforced composite (experimental, SFRC-CAD) and a resin nanoceramic (Cerasmart 270, GC)—were sectioned into standardized specimens (2 mm × 14 mm × 18 mm). Experimental SFRC-CAD blocks were prepared from a mixture of UDMA, TEGDMA (70/30) resins (23 wt%) with short glass fiber (200–300 & Ø7 μm) (25 wt%), and barium glass (52 wt%). Shear bond strength (SBS) was evaluated using two resin-based luting agents: a self-adhesive resin cement (G-CEM ONE, GC) and a short fiber-reinforced flowable composite (everX Flow, GC). Adhesive strategies included primer alone or primer followed by universal adhesive (G2-BOND Universal, GC) application. All resin-based materials used in this study were light-curable or dual-curable. The dental materials used, their manufacturers, and compositions are listed in Table 1.

According to power analysis, the minimum number of test samples required for statistical comparison of test groups was determined as n = 256 (n = 8/group). In calculating the number of test samples per group, the type I error rate (α) = 0.05, effect size (f) = 0.35, and test power (1 − β) = 0.80 were used.

### 2.1. Specimen Preparation

**Figure 1:**
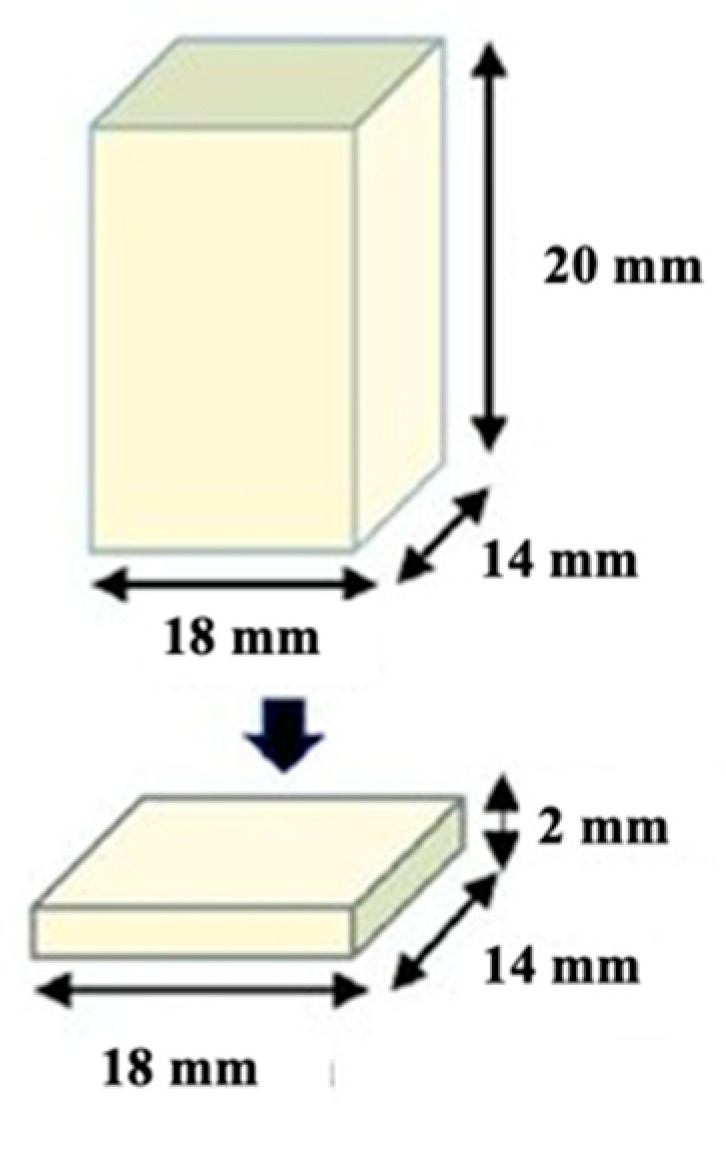
Prepared CAD/CAM restorative block materials (2 mm × 14 mm × 18 mm).

CAD/CAM blocks were sectioned under water cooling using a diamond saw (Secotom-50, Struers, Ballerup, Denmark) to obtain specimens slightly larger than the target dimensions. The specimens were then sequentially ground using silicon carbide papers under water cooling to achieve the final dimensions (2 mm × 14 mm × 18 mm), which were verified using a digital caliper (Figure 1).

**Figure 2:**
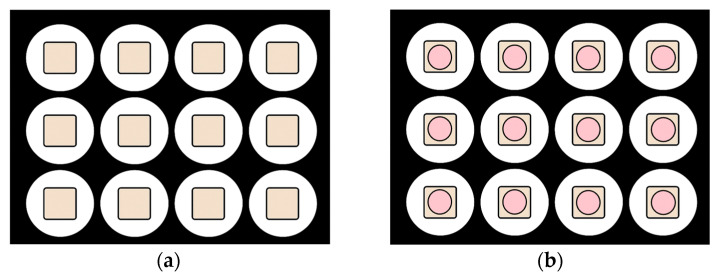
Preparation of acrylic jigs using mold: (**a**) direct curing group; (**b**) indirect curing group with silicone spacer. Beige areas represent the CAD/CAM block material, while pink areas indicate the silicone spacer.

Custom-made acrylic jigs were fabricated to stabilize the specimens during bonding and light-curing procedures. For direct curing, CAD/CAM blocks were embedded centrally in acrylic blocks, leaving the bonding surface exposed. For the indirect curing setup, a silicone spacer (Ø11 mm) was positioned over the CAD/CAM specimen to provide space for indirect light application through the material (Figure 2). Self-cure acrylic resin was poured into the space between the cylinder gap edge and the silicone spacer. After polymerization of the acrylic resin material, the acrylic blocks were removed from the rubber mold.

**Figure 3:**
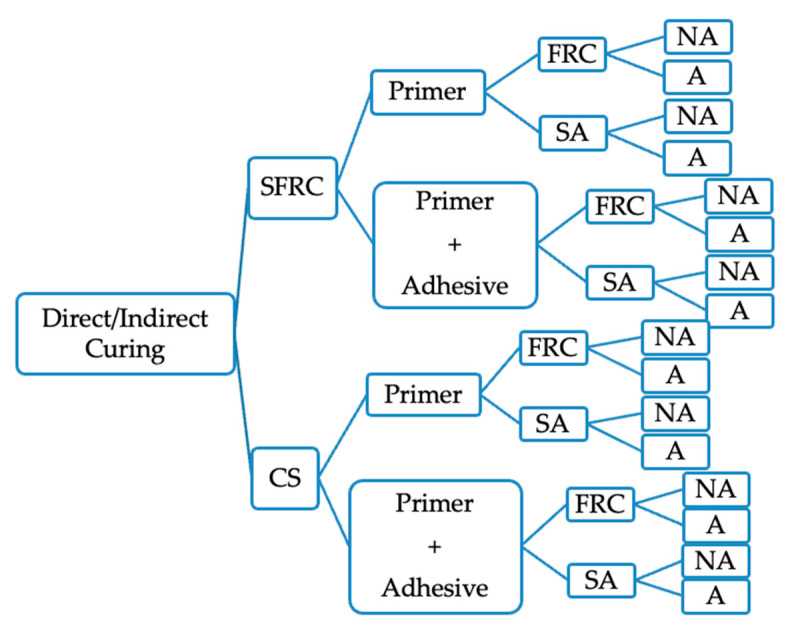
Experiment group flow. SFRC: short fiber composite CAD/CAM block, CS: Cerasmart270, FRC: Short fiber-reinforced resin composite, SA: Self-adhesive resin cement, NA: Non-aged, A: Aged.

Separate specimens were prepared for each curing protocol; no specimen was subjected to both direct and indirect light-curing procedures. Specimens were randomly assigned to direct or indirect light-curing groups, simulating clinical conditions (Figure 3).

A high-intensity monowave LED curing unit (Elipar S10, 3M ESPE, St. Paul, MN, USA) was used for all polymerization steps. The light output (1598.4 mW/cm^2^) was monitored at the beginning of each group using a calibrated radiometer (MARC^®^ system, BlueLight Analytics Inc., Halifax, NS, Canada).

### 2.2. Bonding Protocols

**Figure 4:**
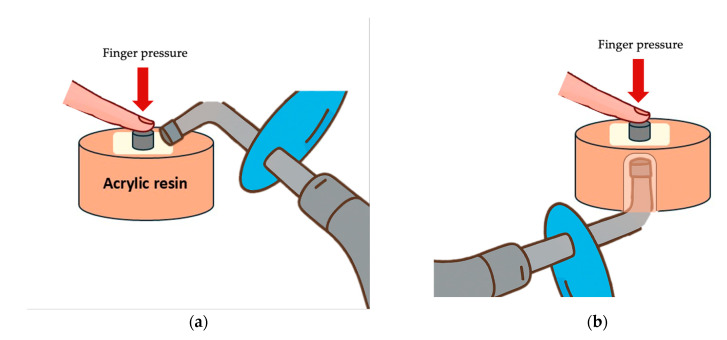
Light curing protocols: (**a**) Direct curing technique, applying light directly to the cement; (**b**) Indirect curing technique, applying light through the CAD/CAM restorative block. Separate specimens were prepared for each curing protocol; no specimen was subjected to both direct and indirect light-curing procedures.

The bonding surfaces of both the CAD/CAM specimens and cobalt-chromium alloy metal cylinders (Ø3.6 mm) were polished with P180 silicone carbide paper and then air-abraded using 50 µm aluminum oxide particles at 2 bar pressure from a 10 mm distance for 10 s. All surfaces were cleaned with oil-free compressed air before bonding. Following surface pre-treatment, the CAD/CAM specimens were divided into two main bonding protocols (Figure 4):

- Primer Only Protocol: G-CEM ONE Adhesive Enhancing Primer was applied to the bonding surfaces using a microbrush for 5 s, followed by gentle air-drying for 10 s. According to their group, either everX Flow (GC) or the G-CEM ONE (GC) was applied to the metal cylinder surface which was then placed onto the CAD/CAM specimen while applying finger pressure;
- Primer + Adhesive Protocol: After primer application and air-drying, G2-BOND Universal (adhesive bottle) was applied for 10 s and air-thinned for 10 s. The adhesive was light-cured for 20 s before the luting materials were applied, and the metal cylinders were positioned as described before.

Excess luting material was removed using a microbrush. All specimens were polymerized according to their group’s assigned light-curing protocol (direct curing or indirect curing). Direct light curing was used as a standardized experimental reference condition to minimize the effect of light attenuation and to evaluate the intrinsic bonding performance of the tested material–adhesive–cement combinations, whereas indirect curing through the CAD/CAM blocks was designed to simulate clinical cementation conditions. Direct curing was performed by applying light directly to the cement, irradiating from four directions for 10 s each (total 40 s). Indirect curing was carried out by applying the curing light through the 2 mm thick CAD/CAM material for 40 s (Figure 4).

### 2.3. Light Irradiance Measurements

The irradiance of the light-curing unit (Elipar S10) was measured using a MARC^®^ resin calibrator under direct exposure and through 2 mm thick CAD/CAM specimens. The mean irradiance under direct exposure was approximately 1598.4 ± 5.9 mW/cm^2^. When measured through 2-mm specimens, the irradiance decreased to approximately 384.8 ± 1.2 mW/cm^2^ for SFRC-CAD, and 211.5 ± 1.5 mW/cm^2^ for Cerasmart 270, indicating substantial light attenuation dependent on material type.

### 2.4. Aging Protocols

After bonding, all specimens were stored in distilled water at 37 °C for 24 h. Half of the specimens were tested immediately (non-aged groups), while the other half were subjected to accelerated hydrothermal aging. The aging procedure was performed by storing the specimens in distilled water at 100 °C for 16 h (UT6060, Heraeus Instruments, Hanau, Germany), following the protocol by Oja et al. (2021) [30,31].

### 2.5. Shear Bond Strength Testing

**Figure 5:**
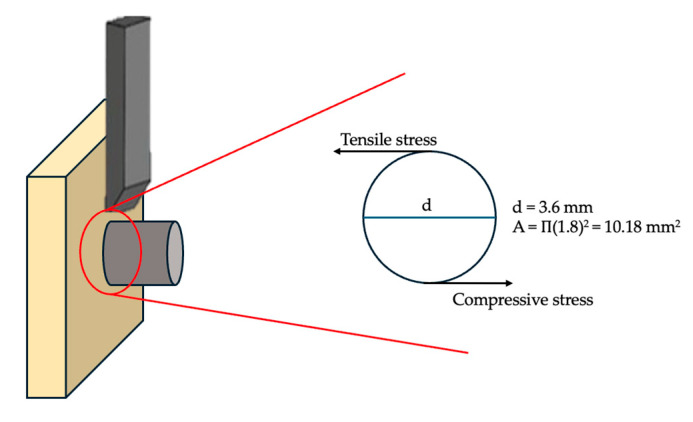
Schematic representation of the shear bond strength (SBS) test setup. Shear stress at the bonded interface is produced by the combined action of compressive and tensile stresses generated during loading. The bonded area is defined by the cross-sectional area of the metal cylinder (Ø3.6 mm).

Shear bond strength tests (SBS) were performed using a universal testing machine (LR30K Plus, Lloyd Instruments, Fareham, UK) at a crosshead speed of 1 mm/min. Specimens were fixed in a custom metal jig, the bonded area was defined by the cross-sectional area of the metal cylinder (Ø 3.6 mm), and shear force was applied parallel to the adhesive interface to ensure consistent geometry and comparable stress distribution across all groups (Figure 5). The maximum force (N) at failure was recorded, and bond strength (MPa) was calculated by dividing this value by the bonding area (mm^2^) using the formula:

$$SBS\;\left(MPa\right)=\frac{Maximum\;Load\;\left(N\right)}{Bonding\;area\;({mm}^{2})}$$

All fractured specimens were examined by visual inspection to determine the mode of failure. Failure modes were classified as adhesive (failure at the restoration–cement interface), cohesive (failure within the restorative material or cement), or mixed (a combination of adhesive and cohesive areas). Statistical analysis was performed using SPSS version 22 (IBM Corp., Armonk, NY, USA). Due to the non-normal distribution of data, the Kruskal-Wallis H test was used to compare SBS values between groups. A significance level of *p* < 0.05 was considered statistically significant.

## 3. Results

Table 2 summarizes shear bond strength (SBS) values as mean ± standard deviation, while median and interquartile range values, which form the basis for statistical interpretation, are provided in the Supplementary Table S1.

The Kruskal–Wallis test revealed a statistically significant difference in SBS values among the 32 tested groups (H = 145.80, *p* < 0.001), indicating that at least one group differed significantly from the others. Therefore, Dunn–Bonferroni post-hoc pairwise comparisons were performed to identify the source of these differences.

Based on median SBS values, groups prepared using direct curing generally demonstrated higher bond strength compared with their corresponding indirect curing counterparts. In addition, aged specimens showed lower median SBS values than non-aged specimens across most experimental conditions.

When individual group comparisons were considered, groups exhibiting the lowest median SBS values were those combining indirect curing and aging, particularly Groups 19, 25, and 27. Post-hoc analysis confirmed that these groups presented significantly lower median SBS values compared with multiple high-performing groups, including Groups 5, 6, 8, 16, 22, 30, and 31 (*p* < 0.05). In contrast, groups incorporating primer followed by universal adhesive application generally demonstrated higher median SBS values than groups treated with primer alone, although not all comparisons reached statistical significance.

In addition to group-wise comparisons, factor-wise pooled analyses were performed using nonparametric methods. Indirect curing resulted in significantly lower median SBS values compared with direct curing (*p* < 0.001). Aging significantly reduced the median SBS relative to non-aged specimens (*p* = 0.006). Application of primer followed by universal adhesive significantly increased median SBS compared with primer alone (*p* < 0.001). When luting materials were pooled, G-CEM ONE demonstrated slightly higher median SBS values than everX Flow (*p* = 0.034). No statistically significant difference in median SBS was observed between SFRC-CAD and Cerasmart 270 when pooled across all experimental conditions (*p* = 0.159). These findings indicate that the curing protocol and adhesive strategy have a significant impact on bonding performance.

**Figure 6:**
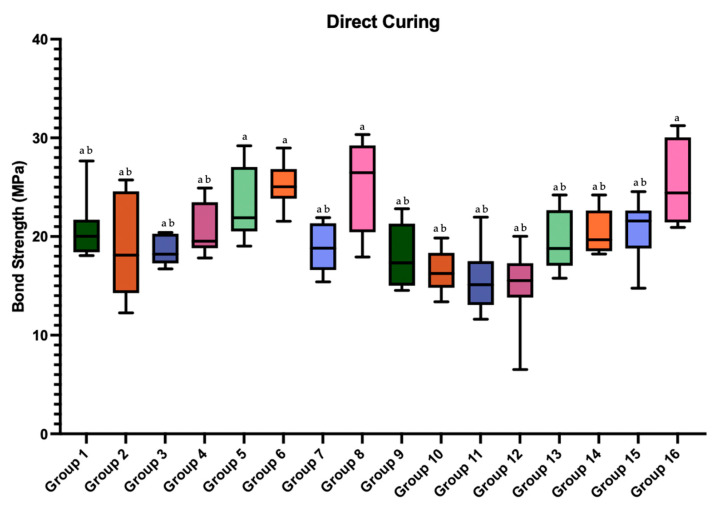
Box plots of the shear bond strength values of the groups prepared with direct curing. Different superscript letters indicate statistically significant differences among groups (*p* < 0.05). Groups sharing at least one letter are not statistically different. Group-wise comparisons were performed using the Kruskal–Wallis test followed by Dunn–Bonferroni post hoc analysis.

**Figure 7:**
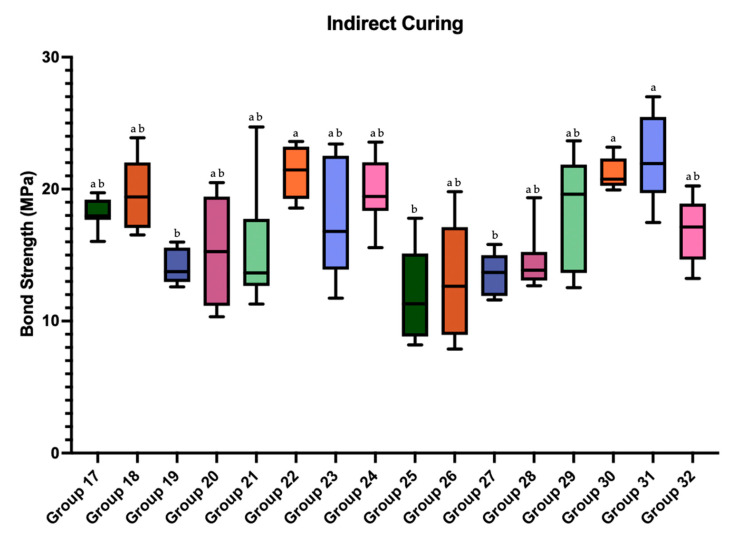
Box plot of the shear bond strength values of the groups prepared with indirect curing. Different superscript letters indicate statistically significant differences among groups (*p* < 0.05). Groups sharing at least one letter are not statistically different. Group-wise comparisons were performed using the Kruskal–Wallis test followed by Dunn–Bonferroni post hoc analysis.

Box plots illustrating the distribution of SBS values for direct and indirect curing groups are presented in Figure 6 and Figure 7, respectively. Superscript letters indicate statistically significant differences among groups (*p* < 0.05).

**Figure 8:**
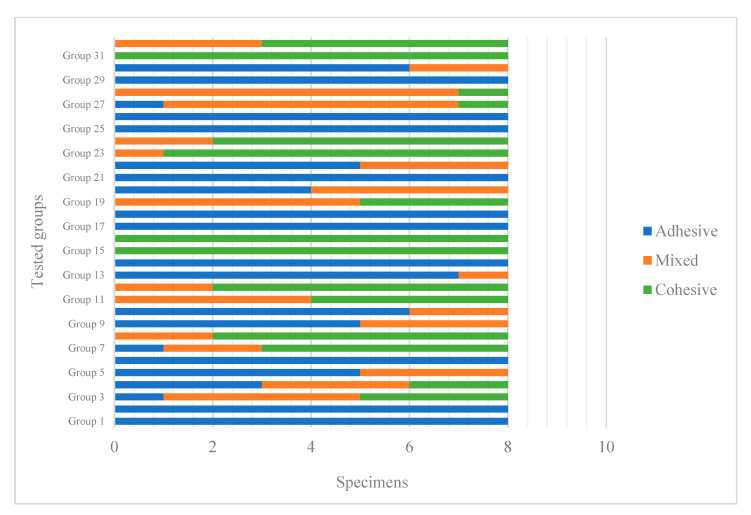
Failure types of the tested groups.

Failure mode analysis revealed predominantly adhesive and mixed failures across the groups, with no cohesive failures observed within the SFRC blocks. Groups demonstrating lower median SBS values (e.g., Groups 19, 25, and 27) exhibited a higher frequency of adhesive failures, whereas groups with higher SBS values showed more mixed failures (Figure 8). The Monte Carlo chi-square test confirmed that the distribution of failure types among groups was statistically significant (*p* = 0.0001).

## 4. Discussion

This study evaluated the influence of material type, bonding protocol, and curing mode on the shear bond strength (SBS) of SFRC-CAD and resin nanoceramic blocks. Although the SBS test is primarily considered a screening method that does not fully reproduce complex intraoral stress conditions, it remains a widely accepted approach for comparative evaluation of adhesive performance under standardized laboratory conditions. The data obtained showed that material type, bonding protocols, and aging processes had an effect on SBS. Accordingly, the null hypothesis of the thesis study was rejected.

CAD/CAM composite blocks are characterized by a highly cross-linked and fully polymerized polymer matrix, which markedly restricts surface dissolution and monomer diffusion required for true interpenetrating polymer network (IPN) formation. Previous studies have demonstrated that bonding to such polymeric substrates differs fundamentally from bonding to freshly polymerized composites, as the dense cross-linked structure limits resin infiltration and, consequently, IPN-based bonding mechanisms [32,33]. Instead, adhesion to CAD/CAM polymer-based materials depends mainly on micromechanical retention achieved by surface roughening and on chemical coupling provided by functional adhesive monomers. This is supported by IPN-related studies showing that effective polymer interdiffusion primarily occurs in semi-IPN or thermoplastic matrices, whereas highly cross-linked networks exhibit limited permeability to low-molecular-weight monomers [34,35,36]. These material-related limitations help to explain the technique sensitivity observed in the present study and highlight the importance of surface treatment and adhesive selection for achieving durable bonding to CAD/CAM polymer-based restorative materials.

Indirect CAD/CAM composites differ from direct composites because polymerization occurs under higher temperature and pressure, producing higher monomer conversion, reduced polymerization shrinkage, and improved mechanical properties [12]. To further improve the mechanical properties of these materials, randomly positioned short fibers are added to the material structure, resulting in short fiber-reinforced CAD/CAM composite resin (SFRC-CAD) restorative blocks with improved toughness and crack resistance [12,37,38,39]. However, the long-term success of indirect restorations is determined not only by material properties but also by reliable adhesion to the tooth structure [40,41]. Key factors in the adhesive bonding of indirect restorations include preparation design, surface conditioning, the type of adhesive used and the application strategy, and the properties of the adhesive cement material [42].

Surface treatments create microporosity and surface irregularities that increase the bond strength between the resin-based adhesive material and the inner surface of the restoration during adhesive bonding [43,44,45]. Both hydrofluoric acid etching and sandblasting have been suggested, but several studies have demonstrated that sandblasting is particularly effective in hybrid ceramics and fiber-reinforced composites [43,44,46]. In this study, all blocks were sandblasted with 50 µm aluminum oxide to optimize bonding conditions, consistent with prior reports.

A review of the literature revealed that, in laboratory conditions, resin-based cylindrical structures were generally preferred in SBS experimental designs [47,48]. In the present study, cobalt-chromium alloy metal cylindrical structures were preferred in order to obtain more realistic results during adhesion evaluation. The reason for this is that the fracture strength value of resin-based adhesive cement material is lower than that of metal alloy fracture strength, which risks cohesive failure within the cement itself rather than true interfacial evaluation. Their higher mechanical strength ensured that failures occurred either at the restoration–cement interface or within the restorative material, allowing a more accurate assessment of bonding performance.

Light attenuation remains a concern in all CAD/CAM materials, as it reduces the degree of conversion of resin cements, particularly with increased thickness and opacity [25,49,50]. Increased thickness or higher filler concentration reduces light transmission and, in turn, limits the polymerization efficiency of underlying resin cements [24,25,51,52]. The degree of polymerization is a critical determinant of the mechanical performance of resin-based materials. In SFRCs, the embedded glass fibers scatter light as it passes through the material, enhancing transmission and permitting application in thicker increments (up to 5–6 mm) compared with conventional particulate-filled composites [53]. This fiber-induced light-scattering behavior may partially compensate for optical attenuation in SFRC-CAD materials, resulting in comparatively improved polymerization of underlying resin cements when compared with particulate-filled CAD/CAM composites under indirect curing conditions.

In conventional in vitro SBS protocols, direct curing is applied to the cement surface to eliminate the influence of light attenuation and to achieve optimal polymerization efficiency. In the present study, direct curing was included as a methodological reference condition rather than a simulation of clinical cementation, allowing the isolated evaluation of the effect of light attenuation on bond strength. Alayad et al. (2021) demonstrated that translucency varied with both material type and thickness, while de Castro et al. (2023) reported a progressive reduction in transmitted light intensity with increasing thickness of CAD/CAM materials [25,28]. Similarly, a systematic review by David-Pérez et al. (2022) showed that light-curing cements polymerized more effectively beneath restorations ≤2 mm compared with dual-cure materials [54]. Consistent with this, the present study found lower SBS values in indirect curing groups compared with direct curing groups. This can be explained by light attenuation through the restorative material, which reduces the degree of conversion of the luting cement and may compromise its mechanical strength, color stability, and biocompatibility. As highlighted by Eichler et al. (2024), the success of polymerization in deeper layers is strongly dependent on the optical transparency of the restorative material [55]. In contrast, direct curing eliminated attenuation and resulted in more effective curing of the cement, which accounts for the higher SBS values obtained in those groups.

Although self-adhesive resin cement material does not require adhesive resin application, manufacturers state that adhesive resin can be used beforehand in necessary cases to increase bonding strength. The universal adhesive tested in this study (G2-BOND Universal) contains 10-MDP, a functional monomer known to chemically interact with hydroxyapatite, forming stable and durable bonds. Previous studies have shown that adhesives containing 10-MDP do not lose their bond strength after long-term aging processes and actually improve their bonding performance over time [17,56,57,58,59]. Martos et al. (2022) compared four 10-MDP-containing adhesives with one without 10-MDP and reported that the bond strength values were higher in the test groups where the adhesive containing 10-MDP was applied [58]. In agreement, our results demonstrated that the application of an adhesive resin after primer generally increased SBS values, although not all differences reached statistical significance.

Although thermocycling is widely used to simulate oral thermal fluctuations and is recommended in ISO standards [60], it primarily induces thermal stress rather than promoting hydrolytic degradation. For highly cross-linked CAD/CAM polymer-based materials, short thermocycling regimens may therefore be insufficient to produce measurable changes in interfacial bonding within a practical experimental timeframe. The hydrothermal aging protocol employed in this study represents an accelerated aging model designed to intensify water diffusion and hydrolytic degradation at the resin–cement interface, allowing a time-efficient evaluation of bond durability in CAD/CAM polymer-based materials [30].

Failure mode analysis further supported these findings. No cohesive failures occurred within the SFRC blocks; instead, adhesive and mixed failures—predominantly adhesive—were observed. The Monte Carlo chi-square test (*p* = 0.0001) confirmed that these differences in failure mode distribution among groups were statistically significant. This pattern can be explained by the fiber length and random orientation of the glass fibers in SFRC, which improve internal fracture resistance and redirect stresses away from the bulk material, thereby helping to arrest and prevent crack propagation, leaving the adhesive interface as the weakest link [13]. Failure mode analysis in the present study was limited to visual inspection, and no detailed fractographic evaluation using microscopic or scanning electron microscopy was performed. Therefore, the lack of high-resolution fractographic analysis represents a limitation of this investigation. Further studies incorporating microscopic characterization of fracture surfaces would be valuable to provide deeper insight into interfacial failure mechanisms.

These findings emphasize that the performance of CAD/CAM indirect restorations is influenced not only by the restorative material but also by the curing protocol and adhesive strategy. Clinicians should be aware that indirect light curing may compromise polymerization due to light attenuation, particularly in thicker or more opaque restorations. These findings suggest that clinicians should prefer direct light access whenever possible or select restorative materials with higher translucency when indirect curing is unavoidable. The use of universal adhesives containing functional monomers such as 10-MDP can improve bonding durability, while surface treatments like sandblasting remain essential to optimize micromechanical retention.

Although this study provides important insight into the bonding behavior of SFRC-CAD and hybrid ceramic materials, several limitations should be acknowledged:

1. This was an in vitro investigation, and laboratory conditions cannot fully replicate the complex oral environment, including temperature fluctuations, masticatory forces, moisture contamination, and oral biofilm activity.
2. Only one surface-conditioning method and a single primer/adhesive system were evaluated; different conditioning protocols or adhesive formulations may yield different outcomes.
3. The aging protocol consisted of short-term hydrothermal aging, which may not fully reflect the long-term degradation mechanisms such as thermocycling, water sorption, or enzymatic degradation.
4. The SBS test provides reliable results but does not replicate the complex multi-directional stresses present during clinical function, and other tests—such as tensile bond strength, three-point bending or fatigue testing—may provide additional insight.
5. Failure mode analysis was limited to visual inspection, and no detailed fractographic evaluation using microscopic or scanning electron microscopy was performed.

Future studies should incorporate long-term aging, alternative surface treatments, additional adhesive strategies, and mechanical fatigue evaluations to better approximate clinical conditions. Additionally, examination of the substrate–cement interface using microscopic or fractographic methods would be beneficial to more precisely identify the site and nature of bond failure in indirect restorations.

## 5. Conclusions

Within the limitations of this in vitro study, the following conclusions can be drawn:

1. Indirect curing resulted in lower SBS values than direct curing, likely due to light attenuation through the restorative material.
2. Applying primer followed by universal adhesive generally increased SBS compared with primer alone.
3. Higher SBS values were observed in groups incorporating adhesive application and direct curing protocols.
4. The use of metal cylinders under finger pressure ensured a more reliable in vitro simulation by more closely approximating clinical film thickness and bonding conditions.

## Figures and Tables

**Table 1:** Summary of materials, manufacturers, and compositions.

Materials	Manufacturer	Composition	LOT Number
CAD/CAM Short Fiber-Reinforced Composite Block	Experimental	UDMA, TEGDMA, short glass fiber (200–300 μm & Ø7 μm), barium glass (77 wt%)	-
Cerasmart 270 (A2 HT)	GC Corporation, Tokyo, Japan	Bis-MEPP, UDMA, DMA, Silica (20 nm), barium glass (300 nm) (71 wt%)	2303156
G-CEM ONE Adhesive Enhancing Primer	GC Corporation, Tokyo, Japan	Ethanol, trimellitic acid, water, 4-methacryloxyethyl phosphate, ester monomer, thiophosphate ester monomer, polymerization initiator	2306061
G2-BOND Universal	GC Corporation, Tokyo, Japan	Primer: 4-MET, 10-MDP, 10-MDTP, dimethacrylate monomer, acetone, water, initiator, filler Adhesive: dimethacrylate monomer, Bis-GMA, filler, photo-activator	2210131
G-CEM ONE Universal self-adhesive resin cement	GC Corporation, Tokyo, Japan	A: Fluoroaluminosilicate glass, methacrylic acid ester, polymerization initiator B: Silica filler, methacrylic acid ester, phosphate ester monomer, polymerization initiator	2210131
everX Flow (Bulk shade)	GC Corporation, Tokyo, Japan	Bis-EMA, TEGDMA, UDMA, short glass fiber (200–300 μm and Ø7 μm), barium-glass (70 wt%)	2302171

**Table 2:** Shear bond strength (SBS) values of the tested groups expressed as mean ± standard deviation.

	Groups	Direct Curing	SBS	Groups	Indirect Curing	SBS
Non-aged	1	SFRC + primer + FRC	20.74 ± 3.16 ^a,b^	17	SFRC + primer + FRC	18.17 ± 1.15 ^a,b^
	2	SFRC + primer + SA	19.12 ± 5.25 ^a,b^	18	SFRC + primer + SA	19.70 ± 2.65 ^a,b^
	3	CS + primer + FRC	18.57 ± 1.52 ^a,b^	19	CS + primer + FRC	14.17 ± 1.31 ^b^
	4	CS + primer + SA	20.79 ± 2.64 ^a,b^	20	CS + primer + SA	15.19 ± 4.16 ^a,b^
	5	SFRC + primer + bond + FRC	23.23 ± 3.67 ^a^	21	SFRC + primer + bond + FRC	15.48 ± 4.38 ^a,b^
	6	SFRC + primer + bond + SA	25.23 ± 2.27 ^a^	22	SFRC + primer + bond + SA	21.21 ± 1.97 ^a^
	7	CS + primer + bond + FRC	18.91 ± 2.46 ^a,b^	23	CS + primer + bond + FRC	17.63 ± 4.46 ^a,b^
	8	CS + primer + bond + SA	25.23 ± 4.61 ^a^	24	CS + primer + bond + SA	19.74 ± 2.51 ^a,b^
Aged	9	SFRC + primer + FRC	18.08 ± 3.20 ^a,b^	25	SFRC + primer + FRC	12.09 ± 3.43 ^b^
	10	SFRC + primer + SA	16.40 ± 2.13 ^a,b^	26	SFRC + primer + SA	13.07 ± 4.37 ^a,b^
	11	CS + primer + FRC	15.54 ± 3.30 ^a,b^	27	CS + primer + FRC	13.55 ± 1.56 ^b^
	12	CS + primer + SA	15.03 ± 3.96 ^a,b^	28	CS + primer + SA	14.56 ± 2.16 ^a,b^
	13	SFRC + primer + bond + FRC	19.51 ± 3.05 ^a,b^	29	SFRC + primer + bond + FRC	18.35 ± 4.31 ^a,b^
	14	SFRC + primer + bond + SA	20.38 ± 2.23 ^a,b^	30	SFRC + primer + bond + SA	21.14 ± 1.19 ^a^
	15	CS + primer + bond + FRC	20.74 ± 3.08 ^a,b^	31	CS + primer + bond + FRC	22.24 ± 3.32 ^a^
	16	CS + primer + bond + SA	25.28 ± 4.1 ^a^	32	CS + primer + bond + SA	16.85 ± 2.39 ^a,b^

SFRC: short fiber composite CAD/CAM block, CS: Cerasmart 270, FRC: Short fiber-reinforced resin composite, SA: Self-adhesive resin cement. Different superscript letters indicate statistically significant differences among groups (*p* < 0.05). Groups sharing at least one letter are not statistically different. Group-wise comparisons were performed using the Kruskal–Wallis test followed by Dunn–Bonferroni post hoc analysis.

## Data Availability

The original contributions presented in the study are included in the article; further inquiries can be directed to the corresponding author.

## Supplementary Materials

The following supporting information can be downloaded at: https://www.mdpi.com/article/10.3390/polym18020160/s1, Table S1: Shear bond strength values of the tested groups.
