# Supplementary material for: Effect of Different Luting Protocols on the Bond Strength of Fiber-Reinforced CAD/CAM Blocks
Source: Polymers (Basel). 2026 Jan 7;18(2):160. doi: 10.3390/polym18020160 (PMC12845148; doi:10.3390/polym18020160)
Supplement: Supplementary file 1 [file polymers-18-00160-s001.zip › polymers-4040448-supplementary.pdf]

Supplementary Table S1. Shear bond strength values of the tested groups.

	Groups	Direct Curing	Median	IQR	Groups	Indirect Curing	Median	IQR
Non-aged	1	SFRC + primer + FRC	20.02	2.75	17	SFRC + primer + FRC	17.96	1.20
	2	SFRC + primer + SA	18.12	9.05	18	SFRC + primer + SA	19.40	4.00
	3	CS + primer + FRC	18.22	2.64	19	CS + primer + FRC	13.75	1.97
	4	CS + primer + SA	19.53	4.07	20	CS + primer + SA	15.26	6.75
	5	SFRC + primer + bond + FRC	21.90	4.69	21	SFRC + primer + bond + FRC	13.66	6.61
	6	SFRC + primer + bond + SA	25.04	2.58	22	SFRC + primer + bond + SA	21.45	3.46
	7	CS + primer + bond + FRC	18.82	4.23	23	CS + primer + bond + FRC	16.79	7.09
	8	CS + primer + bond + SA	26.47	6.41	24	CS + primer + bond + SA	19.44	2.41
Aged	9	SFRC + primer + FRC	17.33	5.06	25	SFRC + primer + FRC	11.32	4.88
	10	SFRC + primer + SA	16.26	2.20	26	SFRC + primer + SA	12.64	5.34
	11	CS + primer + FRC	15.11	3.49	27	CS + primer + FRC	13.68	2.11
	12	CS + primer + SA	15.53	2.91	28	CS + primer + SA	13.86	1.93
	13	SFRC + primer + bond + FRC	18.77	4.42	29	SFRC + primer + bond + FRC	19.62	7.47
	14	SFRC + primer + bond + SA	19.65	2.32	30	SFRC + primer + bond + SA	20.75	0.89
	15	CS + primer + bond + FRC	21.58	3.11	31	CS + primer + bond + FRC	21.93	3.98
	16	CS + primer + bond + SA	24.42	4.13	32	CS + primer + bond + SA	17.13	3.39
